# Comprehensive Analysis of 122 Guinea Fowl Genomes Across Three Continents Delineates Their Domestication and Evolutionary Patterns with Special Reference to India

**DOI:** 10.3390/ijms27072994

**Published:** 2026-03-25

**Authors:** Simmi Tomar, Sheikh Firdous Ahmad, Munish Gangwar, Manoharan Azhaguraja, Alisha Kush, Abha Trivedi, Ravi Kumar Gandham, Ashok Kumar Tiwari

**Affiliations:** 1 ICAR-Central Avian Research Institute, Izatnagar, Bareilly 243122, Uttar Pradesh, India; 2 ICAR-Indian Veterinary Research Institute, Izatnagar, Bareilly 243122, Uttar Pradesh, India; 3 ICAR-Indian Agricultural Research Institute, Dhemaji 787035, Assam, India; 4 Formulation Development Group, Regeneron Pharmaceuticals, New York, NY 10001, USA; 5 Department of Animal Science, M.J.P. Rohilkhand University, Bareilly 243006, Uttar Pradesh, India; 6 ICAR-National Bureau of Animal Genetic Resources, Karnal 132001, Haryana, India

**Keywords:** adaptation, CNV atlas, gene density, genetic diversity, poultry, selection signature, SNP

## Abstract

The guinea fowl (*Numida meleagris*), a thermo-tolerant and disease-resilient poultry species, holds great potential for sustainable poultry production in climate-vulnerable regions. The genomic aspects of this species remain largely understudied. The present study aims to delineate the patterns of domestication and understand the evolutionary dynamics of guinea fowl populations (wild and domestic) across three continents, utilizing whole-genome sequencing data from 122 genomes. The population structure analyses (ADMIXTURE, PCA, phylogeny, F_ST_, LD, and MAF) revealed that Indian guinea fowl (CARI) shared close ancestry with Iranian (IRAD) and Chinese (CHID) domesticated populations while remaining distinct from wild lineages. The runs of homozygosity (ROH) identified 49,088 segments, with short fragments (ROHs) preponderant in Indian and domestic populations, reflecting historical inbreeding and effects of domestication cum selection. Copy number variation (CNV) analysis revealed 105,178 CNVs concatenated into 40,067 CNV regions (CNVRs) across 11 populations, establishing the first CNV atlas for guinea fowl at the global level. Gene annotation of overlapping ROH and CNVRs revealed 1080 common candidates across Asian guinea fowl populations, i.e., the Indian guinea fowl (CARI), IRAD, and CHID, including *FOS*, *EPAS1*, *CD74*, and *CSF1R*. These genes have earlier been associated with immune regulation, stress response, and thermal adaptation. Selection signature scans, integrating intra-population (iHS) and inter-population (XP-EHH) approaches, uncovered genes under positive selection linked to immune response (like *BCL11B*, *IL18*, and *GPC3*), thermo-tolerance (like *TRPV4* and *BAG3*), lipid metabolism (like *AACS* and *ELOVL4*), and pigmentation (*BCO2*). These signatures highlight the molecular basis of resilience in guinea fowl and their potential to withstand climate-induced stresses. This study presents the first global CNV atlas for guinea fowl and provides the first comprehensive genomic characterization of the Indian domestic population, integrating ROH, CNV, and selection signature analyses. It offers a comprehensive assessment of guinea fowl genomes (wild and domesticated) across three continents, offering insights into domestication, evolutionary dynamics, and the genetic basis of their adaptation and resilience.

## 1. Introduction

The helmeted guinea fowl (*Numida meleagris*), a member of the Galliformes order and Numididae family, is native to sub-Saharan Africa [1]. Domestication of the species is believed to have occurred approximately 2000 years ago in regions such as Mali and Sudan [2], where local and wild populations often interbreed [1]. Since domestication, guinea fowl have spread globally, evolving into diverse populations with varying morphology, physiology, and behavior. This diversity has resulted from genetic adaptation along with the effects of natural and artificial selection [3,4,5]. They have adapted to a wide range of environments, including tropical, Mediterranean, and arid regions of Africa, as well as temperate and subarctic regions of Europe [3]. The guinea fowl and geese together make up 1.99% of the global poultry population, compared to chickens (91.18%), ducks (5.60%), and turkeys (1.22%) [6]. France leads the global guinea fowl population, while Italy, Belgium, and Scandinavian nations also contribute significant proportions [7].

Guinea fowl were introduced to India in the seventeenth century. They are valued for their meat, eggs, and ornamental feathers. In rural areas, they are mostly kept as free-ranging scavengers rather than being commercially farmed. Popular varieties include Pearl, Lavender, and White [8]. Additionally, improved varieties like ‘GUNCARI’ have been recently developed. They exhibit a marketable body weight of around one kg at 12 weeks and produce 100–120 eggs per laying season [8]. Despite their agricultural and scientific significance, genetic research on guinea fowl remains limited as compared to chickens, with only a few studies addressing genetic diversity [9,10]. Previous studies on the genetic diversity of guinea fowl have been undertaken using mitochondrial DNA [9,10] and microsatellite markers [11,12], revealing an absence of genetic structuring in domestic HGF populations.

Genetic diversity encompasses the total genetic variation within and among species, serving as a foundation for adaptability to environmental changes [13,14]. Analyzing genetic diversity provides insights into the origin of breeds, their evolutionary history, and unique traits within the gene pool, offering valuable guidance for conservation and breed improvement programs [15]. Additionally, analyzing runs of homozygosity (ROH), long stretches of homozygous DNA segments inherited from common ancestors, may provide valuable insights into past inbreeding and selection pressures [16]. ROHs can complement selection signature analyses by identifying genomic regions under selection pressure that contribute to adaptation and resilience in challenging environments [17]. Selection signatures are genomic footprints left by natural or artificial selection, reflecting changes in the frequency of selected loci and linked neutral regions. These can be used to pinpoint genes under selection [18].

Single-nucleotide polymorphism (SNP) markers have gained immense significance in the field of population genomics due to their abundance and near-uniform genome-wide distribution and are widely used for detecting population structure and breed classification [19], besides the assessment of genetic diversity [20] and selection signatures [21]. Likewise, copy number variations (CNVs), exhibiting deletions or duplications as compared to a reference genome, significantly impact gene dosage and structure and consequently influence phenotype (in terms of economic traits) [22]. CNVs have been reported to contribute both functional and evolutionary variations [23] and have been studied across multiple species, including cattle [24,25], buffaloes [26,27], pigs [28,29], and chickens [30,31].

Elucidation of SNP markers on a genome-wide scale mainly relies on selected approaches, including double-digest Restriction-site Associated DNA (ddRAD) sequencing, SNP chips, or whole-genome sequencing (WGS). The ddRAD, though cost-effective and used across a few poultry species [32], involves reduced representation and faces limitations with larger genomes. SNP chips, based on a preselected SNP set, may miss rare variants and distort allele frequency estimates due to ascertainment bias [33]. Moreover, the SNP arrays are not available for all poultry species. WGS overcomes these complexities by capturing both common and rare variants, offering more accurate and unbiased insights into various genomic parameters. As the threat of climate change continues to grow, the adaptability of poultry species such as guinea fowl gains vital significance [34]. Genomic studies in livestock species such as chickens [35,36], cattle [37], sheep [38], and goats [39] have demonstrated clear genetic adaptations to hot and arid environments. In contrast, guinea fowl, known for their naturally higher thermo-tolerant and disease-resilient abilities as compared to chickens, have yet to be thoroughly explored through genomic studies [40]. The genetic basis of their adaptation to harsh environments remains largely unexplored. Furthermore, identifying selection signatures across various comparisons involving guinea fowl could help reveal key genes linked to thermo-tolerance and disease resilience.

No study has ever reported the differentiation of the Indian guinea fowl population on a genomic scale from global counterparts covering various aspects, including population structure, ROH, fixation index (F_ST_), CNV, and selection signature analyses. Therefore, the present study aimed to investigate the genome-wide differentiation of Indian guinea fowl from global domesticated and wild populations using WGS data and find selective sweeps linked to thermo-tolerance and disease resilience. Alongside SNP-based analysis, a read-depth approach was employed to establish the first-ever reported genomic atlas of copy number variation in global guinea fowl populations in relation to gene density across their genome. It shall help uncover structural variations associated with thermal adaptation and immune response.

## 2. Result

Molecular genetic analysis using genome-wide SNP markers derived from WGS data across multiple guinea fowl populations offers a valuable opportunity to identify the genetic variants and biological pathways involved in environmental adaptation and innate disease resilience. The present study aimed to uncover the in-depth insights into the evolution and adaptation of Indian guinea fowl populations compared to their African, Asian, and European counterparts, using advanced bioinformatics tools and population genomics approaches on WGS data across 11 different populations including domestic, i.e., Indian (CARI, *n* = 6), Iran (IRAD, *n* = 23), China (CHID, *n* = 6), Italy (ITAD, *n* = 16), Hungary (HUGD, *n* = 12), Sudan (SUDD, *n* = 10), Kenya (KEND, *n* = 8), Nigeria (NIGD, *n* = 14); and wild i.e., Nigeria (NIGW, *n* = 12), Kenya (KENW, *n* = 12), and Sudan (SUDW, *n* = 11) cohorts.

### 2.1. Population Structure Assessment

**Figure 1:**
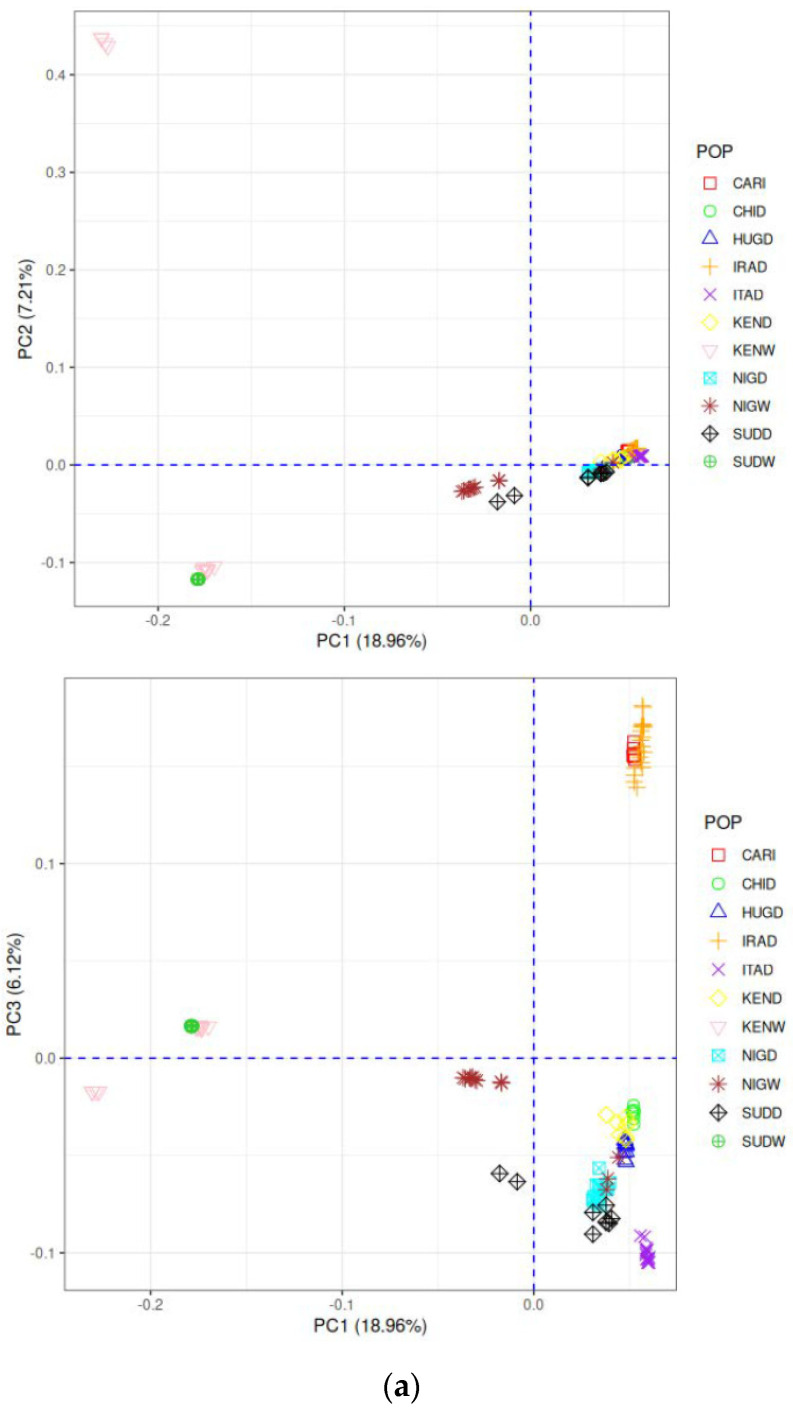

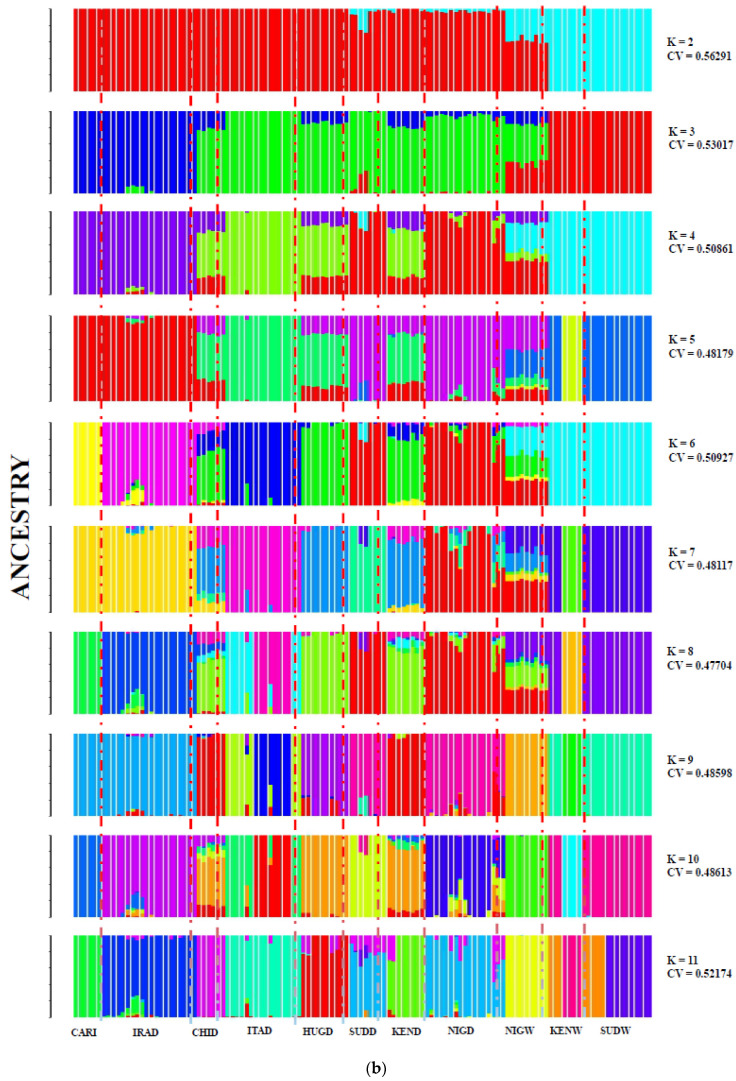

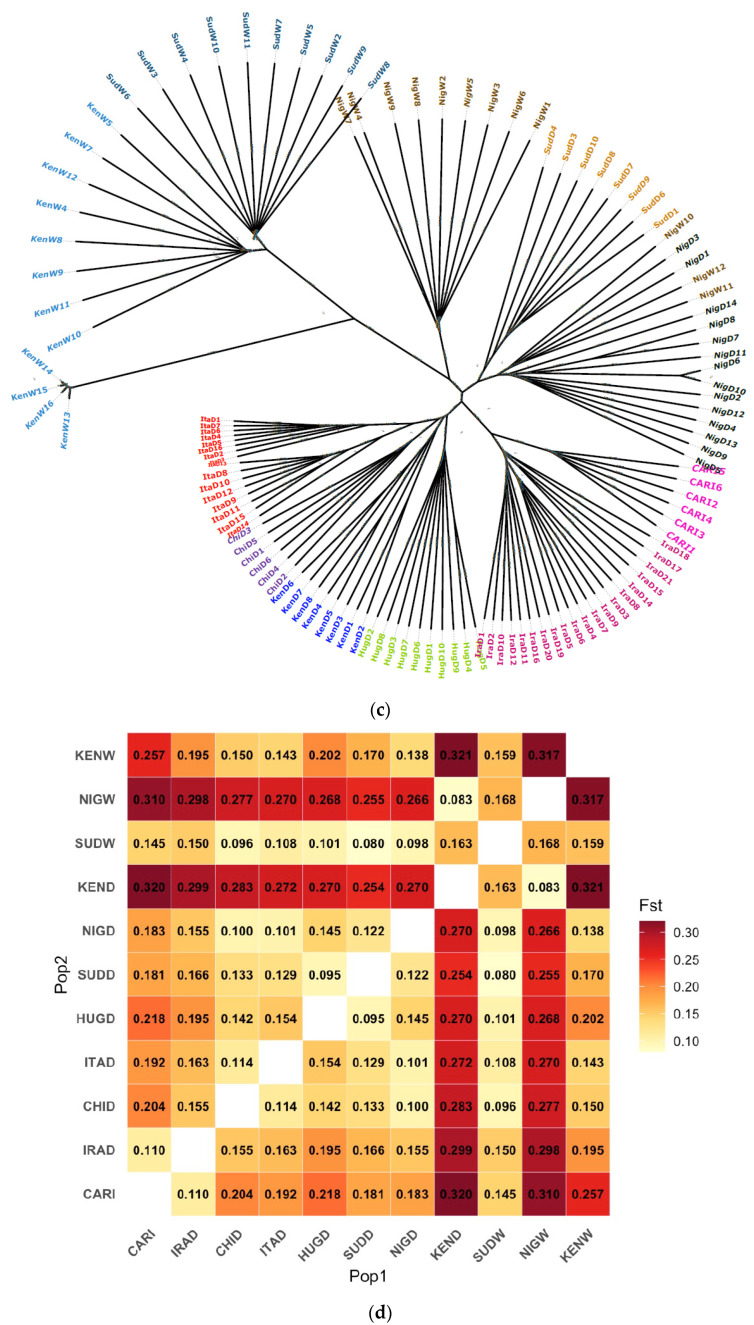

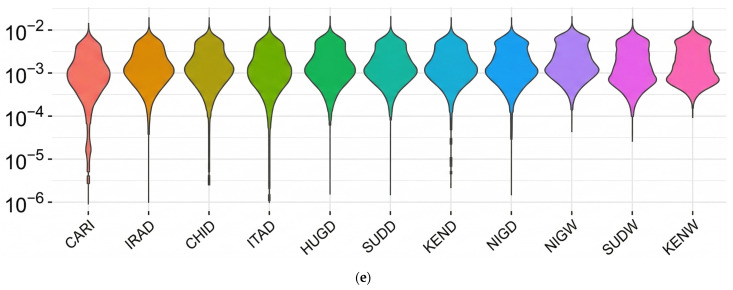
(**a**) PCA plot showing genetic clustering of guinea fowl population based on whole genome SNP data (PC1 vs. PC2 and PC1 vs. PC3). (**b**) ADMIXTURE bar plots at K = 2–11 with cross-validation errors illustrating population structure and ancestry proportions of 11 guinea fowl populations. (**c**) Neighbor-joining tree based on genetic distance depicting the relation between 11 guinea fowl populations. (**d**) Genetic differentiation (pairwise FST) among domestic and wild guinea fowl populations across 55 comparisons (symmetric matrix). (**e**) Violin plot depicting the distribution of nucleotide diversity among domestic and wild guinea fowl populations.

On population structure analysis using PCA and ADMIXTURE, eight samples (three IRAD, two HUGD, two SUDD, and one SUDW) were excluded from downstream analysis due to their stratification with other clusters. The study obtained over eight million (8,622,463) high-quality SNPs from 122 individuals using variant calling and quality control, with an average genotyping rate of 98.89%. The chromosome-wise distribution of markers along the guinea fowl genome is presented in Supplementary File S1. The PCA revealed genetic relationships among 11 guinea fowl populations, providing insights into their population structure (Figure 1a,b). The eigenvectors of PC1, PC2, and PC3 in guinea fowl populations accounted for 32.29% of the total genetic variation, with individual contributions of 18.96%, 7.21%, and 6.12%, respectively. The ADMIXTURE analysis showed distinct clustering patterns in domestic and wild guinea fowl populations. At K = 2, domestic and wild guinea fowl populations stratified into two main clusters, with Indian guinea fowl (CARI) clustering with the domestic cohort (Figure 1b). The Indian and Iranian guinea fowl started stratifying at K = 3, while the Indian guinea fowl (CARI) showed exclusive clustering, starting at an early value of K = 6. At K-values 3–5, domestic populations began to diverge into subgroups, with Indian guinea fowl (CARI) closely aligned to IRAD, CHID, and wild populations showing initial regional clustering. At a K-value of 6–7, further sub-structuring emerged, with ITAD, HUGD, and SUDD forming distinct clusters, wild populations separating more clearly by region, while the Indian guinea fowl (CARI) formed strong clustering to IRAD with minor shared ancestry from NIGW. At K = 8, finer sub-structuring was observed, separating distinct domestic lineages and revealing regional differentiation among wild populations. At K values of 10 and 11, each population formed distinct genetic clusters, indicating minimal recent gene flow (Figure 1b). The cross-validation error was minimal for the K value of 8.

Phylogenetic tree analysis (Figure 1c) revealed the stratification of 11 guinea fowl populations into two major clades. Domestic populations, including Indian guinea fowl (CARI), formed a distinct clade with genetic similarity to IRAD. On the other hand, CHID, ITAD, HUGD, and SUDD clustered within the domestic clade while forming their sub-branches. The wild populations (NIGW, KENW, and SUDW) formed separate clades with further sub-clustering corresponding to their geographic origins.

The pairwise F_ST_ estimates among the 11 guinea fowl populations and 55 comparisons are presented in Figure 1d. The Indian guinea fowl (CARI) showed low differentiation from other domestic populations, ranging from 0.110 (IRAD) to 0.218 (SUDD).

The MAF analysis across the guinea fowl population revealed a significant proportion (more than 50%) of the makers scored MAF levels within the first bin (bin I: MAF > 0.50) for five populations, i.e., CARI, IRAD, CHID, ITAD, and SUDD. The estimate was 66.44% in Indian guinea fowl (CARI). The bin II (0.10–0.20) accounted for 7.56% (CARI) to 18.45% (NIGW), while the bin III (0.20–0.30) proportions varied from 6.71% (SUDD) to 20.49% (NIGW). The highest MAF category (>0.40) ranged from 10.81% (NIGD) to 15.92% (SUDW). Table 1 depicts the empirical distribution of average minor allele frequency (MAF) across five different bins and LD estimates in 11 guinea fowl populations.

Nucleotide diversity (π) ranged from 0.14% in Indian guinea fowl (CARI) to 0.25% in NIGW. Wild populations (NIGW 0.25%, KENW 0.22%, and SUDW 0.21%) showed higher diversity, while domestic populations (CARI 0.14%, ITAD 0.17%, and CHID 0.19%) exhibited lower diversity (Figure 1e).

### 2.2. Runs of Homozygosity (ROH)

**Figure 2:**
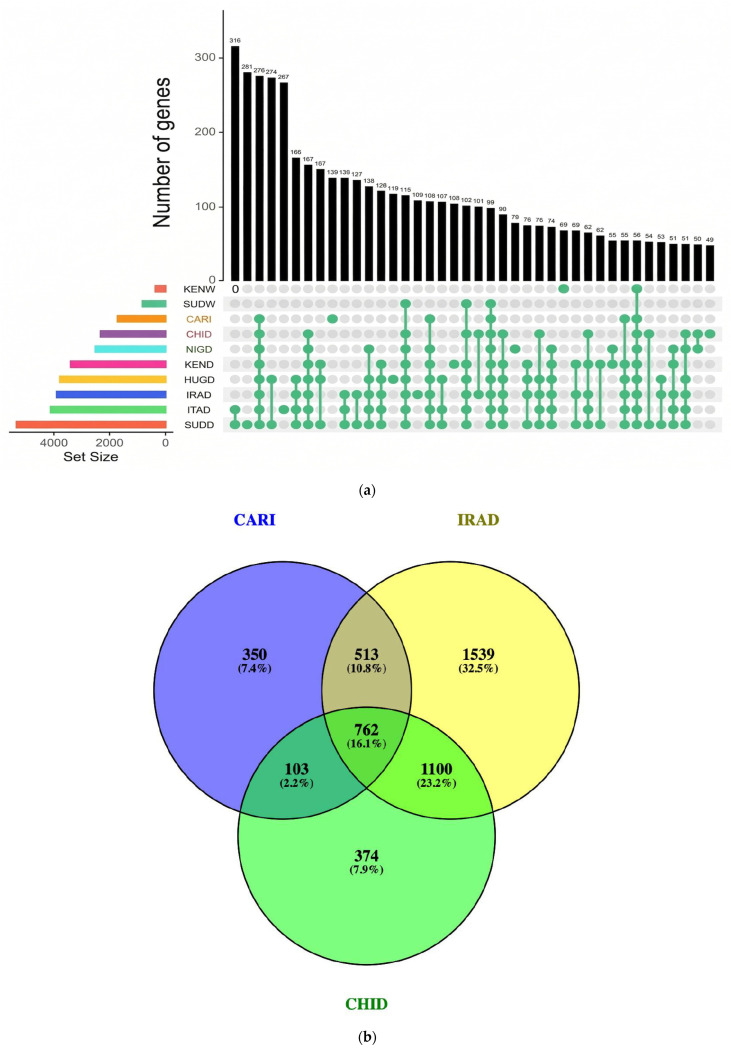
(**a**) Upset diagram showing overlap of genes among 10 guinea fowl populations with ROH exceeding 1500 kb. (**b**) Venn diagram showing the overlap of genes among the Asian guinea fowl population, i.e., CARI, IRAD, and CHID, with ROH exceeding 1500 kb.

The ROH analysis showed substantial variation among 11 guinea fowl populations, with total homozygous fragments ranging from 337 in SUDW to 10,552 in IRAD, while the Indian guinea fowl (CARI) scored 2983 segments. Mean ROH length was lowest in SUDW (363.69 kb) and highest in SUDD (719.08 kb), whereas Indian guinea fowl (CARI) averaged 547.62 kb (Table 2). The average number of ROH fragments per individual was highest in ITAD (604.88), followed by SUDD (598.38), and lowest in SUDW (33.7). The ROH density was consistent across most populations (0.18–0.20), except for higher values in NIGW (0.23) and SUDW (0.34), indicating denser SNP coverage in wild populations. Short ROHs (<500 kb) dominated across different populations, peaking in wild lineages (>89%) and occurring at moderate levels in domestic cohorts (47–67%). The largest single ROH was detected in SUDD (8.84 Mb; 0.84% of the genome), followed by ITAD (7.82 Mb; 0.76% of genomic coverage) and KEND (8.02 Mb; 0.74% of genomic coverage). Most ROHs were located on the first five autosomes, relating to their larger size, while chromosomes 8, 15, 18, 23, and 31 harbored the lowest counts. The number of SNPs per ROH increased along the ROH length. In this study, 49,088 ROHs were identified, including 2005 runs exceeding 1500 kb, which harbored 7213 protein-coding genes (72.62%) and 2719 non-protein-coding genes (27.38%). Additionally, several genes harbored were found to be common across populations (Figure 2a), with 763 genes identified as shared among the Indian guinea fowl (CARI), IRAD, and CHID populations within SNP regions located in ROHs (Figure 2b). The details on genes harbored under ROH fragments across these populations are presented in Supplementary File S2.

### 2.3. CNV and CNVR Map of Global Guinea Fowl Populations

A total of 105,178 unique CNV events were scored across 11 guinea fowl populations, comprising 100,551 deletions and 4628 duplications, with a mean of 862.11 CNVs per animal. The CNV lengths ranged from 1.2 to 8494.9 kb. The highest CNV counts were observed in HUGD (16,546), followed by IRAD (14,765), KEND (12,341), and ITAD (12,227), while Indian guinea fowl (CARI) had the lowest number of CNVs (2672). HUGD exhibited the highest number of deletion-event CNVs (16,159), and ITAD showed the highest duplication events (1009). Overall, CNVs revealed an average length of 352.34 Mb. The majority of CNVs were located on the first six autosomes, consistent with their larger size, whereas chromosomes 8, 9, 15, 18, 23, and 31 harbored the lowest counts.

**Figure 3:**
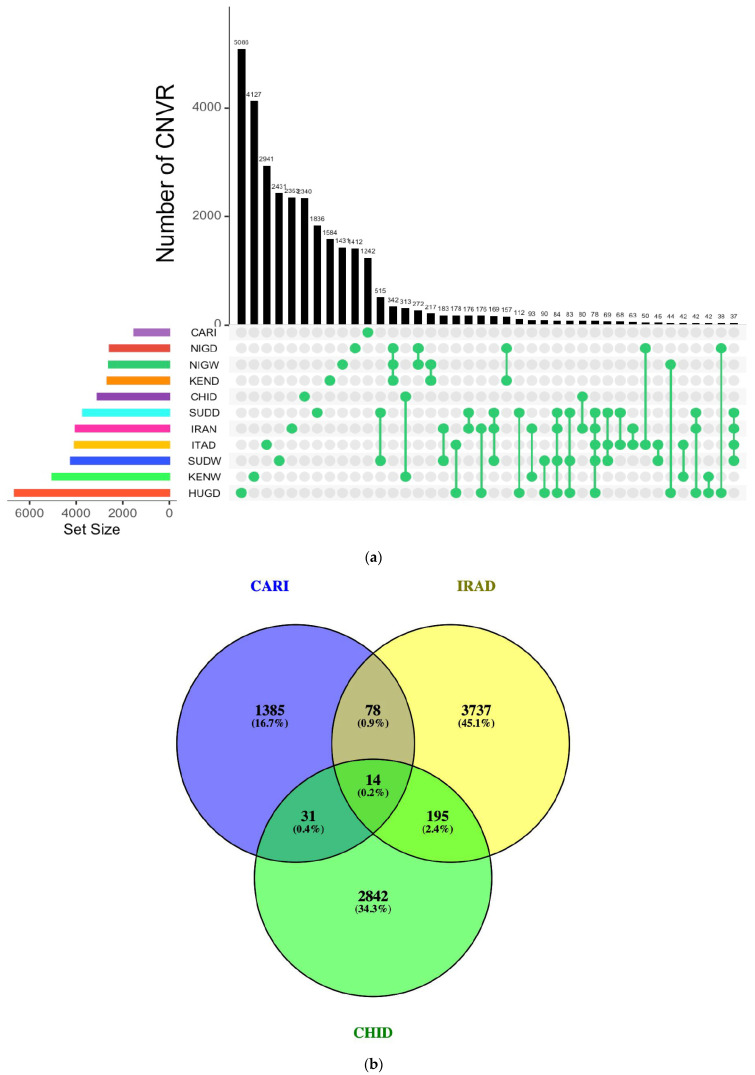

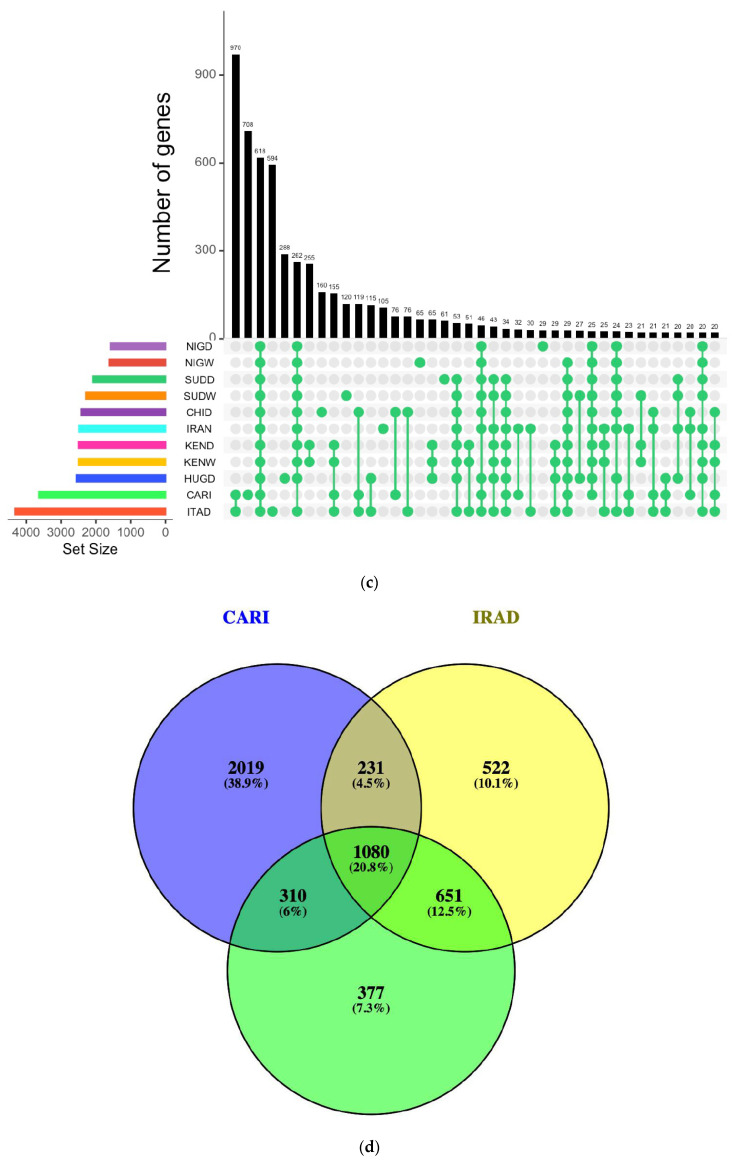
(**a**) Overlapping CNVR among 11 guinea fowl populations. (**b**) Common CNVR among the Asian guinea fowl population, i.e., CARI, IRAD, and CHID. (**c**) Overlapping CNVR genes among 11 guinea fowl populations. (**d**) Common CNVR genes among the Asian guinea fowl population, i.e., CARI, IRAD, and CHID.

**Figure 4:**
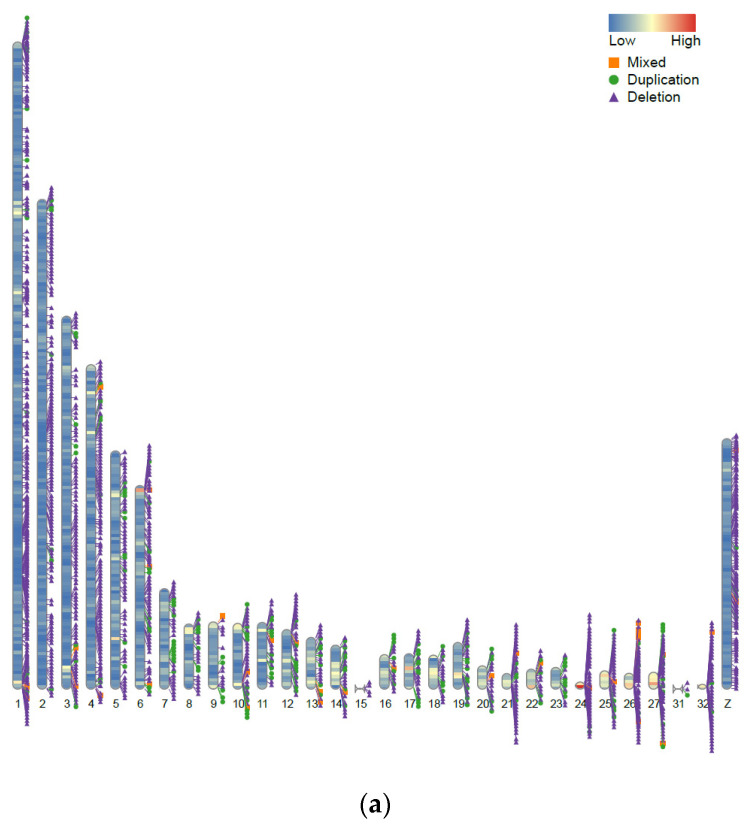

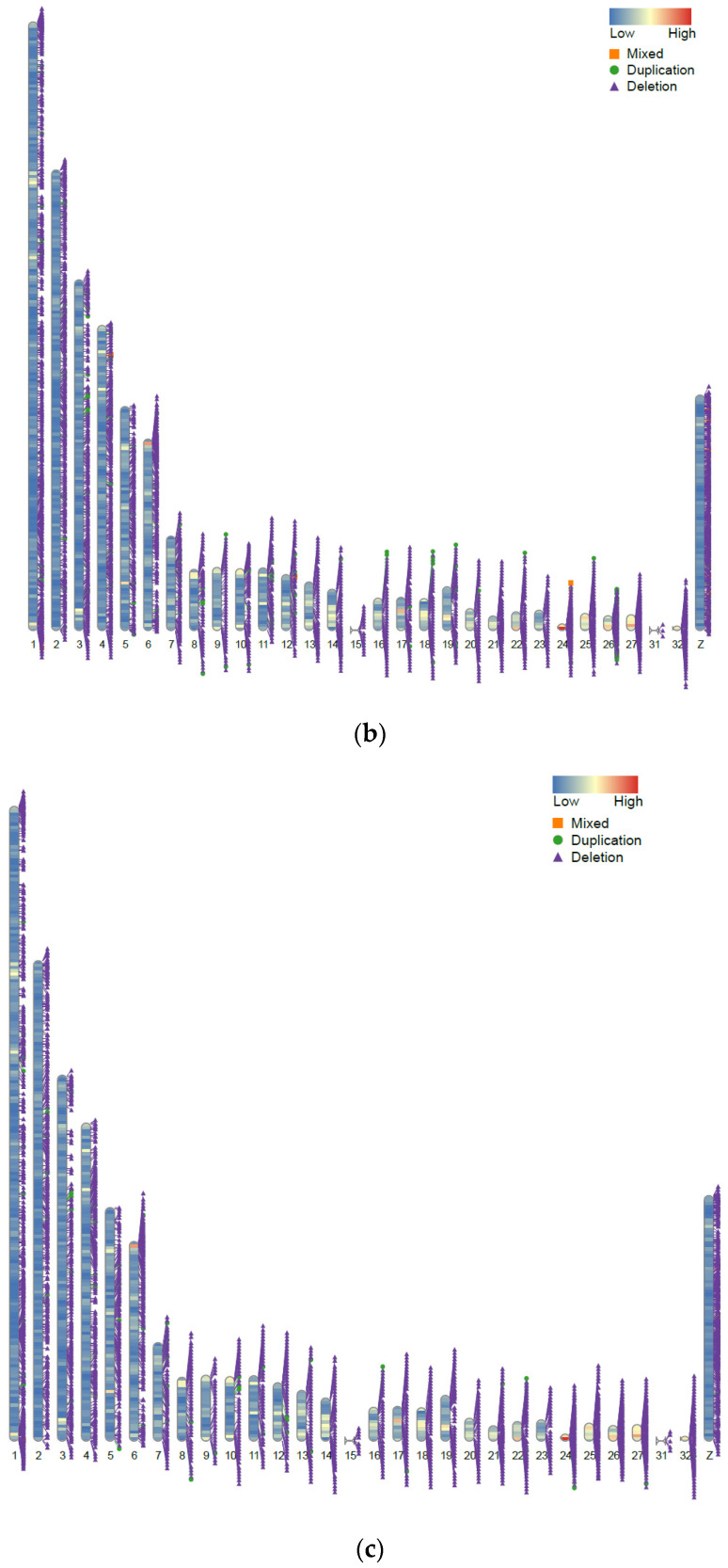
(**a**) CNVR map of the Indian guinea fowl (CARI) population in relation to gene density. (**b**) CNVR map of the Iranian domestic (IRAD) guinea fowl population in relation to gene density. (**c**) CNVR map of the Chinese domestic (CHID) guinea fowl population in relation to gene density.

On concatenation, 40,067 CNVRs were scored, consisting of 38,072 losses (deletions), 1767 gains (duplication), and 228 mixed types. Loss-type CNVRs were most frequent in HUGD (6496), KENW (4789), SUDW (4036), and ITAD (3634), while the lowest number was recorded in Indian guinea fowl (CARI), i.e., 1266. Gain-type CNVRs were most in ITAD (336), KENW (226), and IRAD (221) and lowest in Indian guinea fowl (CARI), i.e., 8. Mixed-type CNVRs were present in all breeds, with the highest counts in ITAD (93), CARI (34), and IRAD (20). Table 3 depicts the summary statistics of CNVR counts, types, and genomic coverage across 11 guinea fowl populations. Figure 3a depicts CNVRs common to all populations, while Figure 3b summarizes those shared among Indian guinea fowl (CARI), IRAD, and CHID. An atlas of mapped CNVRs in relation to gene density for these three populations is depicted in Figure 4a–c, with others in the Supplementary File S3.

The dataset on the CNVR was mined against the guinea fowl gene database. Among 40,067 CNVRs identified in the present study, 31,034 (77.46%) did not harbor any genes. A total of 7274 (80.53%) gene-containing CNVRs harbored protein-coding genes, and 1759 (19.47%) harbored non-protein-coding genes or regulatory elements. The details on the genes harbored under CNVR across 11 populations have been presented in Supplementary File S4.

### 2.4. Selection Signatures

The study utilized a dual complementary approach to investigate the genetic variants underlying adaptation in Indian guinea fowl (CARI), assessing selection signatures both within and between populations.

### 2.5. Intra-Population iHS Selection Signals Within Indian Guinea Fowl (CARI)

**Figure 5:**
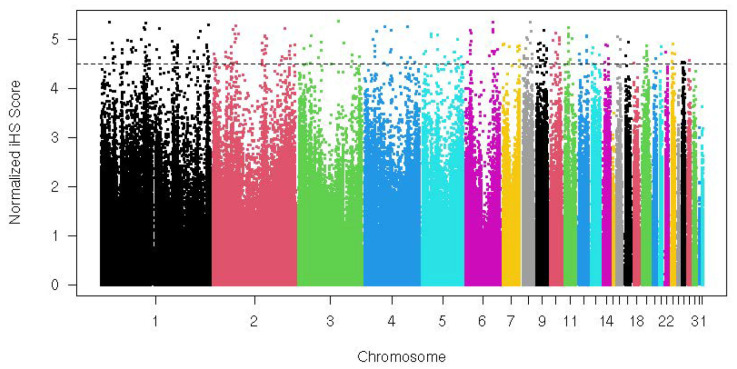
Manhattan plot showing adaptation footprints within Indian guinea fowl in terms of distribution of iHS values.

Intra-population selection signature analysis using the iHS method identified several SNPs under positive selection in Indian guinea fowl (CARI). These SNPs were distributed across multiple autosomes of the guinea fowl genome (Figure 5). Several genes overlapping with the identified SNPs (selective sweeps) within a 100 kb window include *ANKRD2*, *BCL11B*, *CDH13*, *GPC3*, *IGDCC4*, *IGSF11*, *IL18*, *IRS4*, *TCF12*, *TET3*, and *TRPV4.*

### 2.6. Inter-Population Selective Sweeps

The study compared the Indian guinea fowl (CARI) genome with other domestic guinea fowl populations (IRAD, CHID, ITAD, HUGD, SUDD, KEND, and NIGD) to identify population-specific selection signatures. The Indian guinea fowl (CARI) genome served as the query, with other domestic populations as references. A 100 kb genomic window was used to assess SNPs in selective sweeps, mostly located in gene-dense regions.

### 2.7. XPEHH Selection Signals: Indian Versus Domestic Guinea Fowl

**Figure 6:**
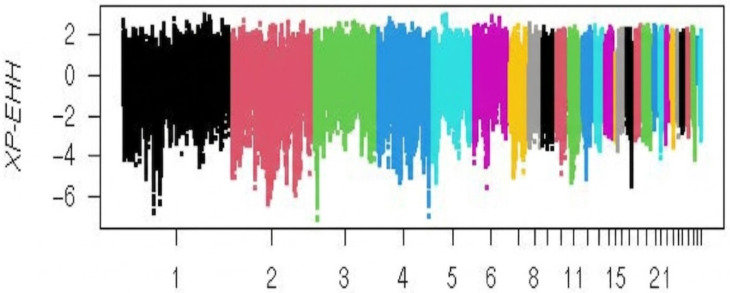
Manhattan plot showing adaptation footprints on comparison of the Indian guinea fowl with the domestic guinea fowl population.

In this comparison, several SNPs associated with selection signals were identified in both groups (Figure 6) and located near various protein-coding genes linked to enhanced adaptability. In the Indian guinea fowl (CARI) cohort, 251 genes were found within selective sweep regions, whereas 86 genes were located near such regions in the domestic guinea fowl population.

## 3. Discussion

Guinea fowl is a vital animal genetic resource that significantly impacts rural and tribal communities worldwide, including India. Their unique characteristics, including thermotolerance and disease resilience to common poultry diseases, make them a promising species for sustainable poultry production in climate-vulnerable regions [41,42]. In the population structure analysis, the Indian guinea fowl (CARI) was closely related to domesticated lineages, i.e., IRAD, CHID, HUGD, and ITAD. In contrast, wild guinea fowl populations, i.e., NIGW, SUDW, and KENW, clustered distinctly from the domestic group along PC2 and PC3 axes (Figure 1a). In concurrence with the present study, Vignal et al. [1] reported a similar genetic distinction between wild and domestic guinea fowl populations on PCA. The results of population structure analysis complemented each other and revealed genetic closeness of populations in line with their domestications and geographical distribution across continents. Indian guinea fowl population (CARI) showed closeness with Asian populations, i.e., IRAD and CHID. This may be indicative of their shared ancestry. The wild guinea fowl populations showed early and clear genetic separation from each other as compared to domestic cohorts. Limited gene flow was also indicated between the Indian guinea fowl (CARI) and Nigerian wild (NIGW) populations. The closest relationship of Indian guinea fowl (CARI) in terms of F_ST_ estimates was revealed for IRAD (F_ST_ = 0.110), followed by CHID (F_ST_ = 0.204) and HUGD (F_ST_ = 0.192), indicating strong genetic similarity among domesticated guinea fowl. Adeola et al. [9] reported a lower F_ST_ (0.035) from mitochondrial D-loop sequence data within seven Nigerian guinea fowl populations compared to the current study. Among wild guinea fowl, Indian guinea fowl (CARI) showed genetic similarity with NIGW (F_ST_ = 0.145), suggesting partial shared ancestry or gene flow. However, this needs further research evidence for a conclusive inference. The F_ST_ values between domestic and wild guinea fowl were generally higher, reflecting substantial genetic divergence, with the lowest differentiation observed between KEND and NIGW (F_ST_ = 0.098) and the highest between ITAD and SUDW (F_ST_ = 0.321). These findings suggested that although occasional gene flow may have occurred, domestic and wild populations remain largely distinct, consistent with the PCA, ADMIXTURE, and NJ-tree results. Consistently low F_ST_ values among domestic populations reflected their close genetic relatedness. Despite divergence between domestic and wild populations, lower F_ST_ values, especially between KEND and NIGD (0.032), were indicative of shared ancestry or historical gene flow between them. SUDD was the most diverged population among 11 cohorts with consistently higher F_ST_ values, followed by KENW. Similarly, KEND was the least diverged population. Overall, the F_ST_ estimates among different comparisons complemented the results of population structure and admixture analysis, revealing the influence of geographical proximity and historical events on population differentiation. Low-frequency alleles (MAF < 0.10) dominated most guinea fowl populations, with CARI showing the highest proportion (66.44%). A similar trend was observed for the estimated r^2^ values, which ranged from 0.353 in NIGW to 0.689 in the Indian guinea fowl (CARI). Wild populations, i.e., NIGW (r^2^ = 0.353), SUDW (r^2^ = 0.363), and KENW (r^2^ = 0.397), showed the lowest LD estimates, indicative of larger effective population sizes and higher historical recombination rates. In contrast, higher LD values were recorded in CARI (r^2^ = 0.689), ITAD (r^2^ = 0.606), and IRAD (r^2^ = 0.563), suggesting smaller effective population sizes or the influence of recent selection. In concurrence with the present study, Vignal et al. [1] similarly reported that LD decay occurred at a faster rate in wild guinea fowl populations as compared to the domestic cohorts. The highest proportions of bin-I markers in CARI were indicative of the presence of rare alleles with genetic structure consistent with strong selection pressure or a recent bottleneck or smaller effective population size (Ne). Wild guinea fowl populations revealed a lower proportion of rare alleles and lower LD estimates, suggesting a larger effective population size and higher recombination rates compared to the domesticated counterparts. The skewed MAF and LD distribution in guinea fowl populations was shaped by their demographic history and can be taken as a distinguishing feature among them. Vignal et al. [1] reported higher nucleotide diversity in wild guinea fowl populations as compared to the domestic guinea fowl cohorts. The wild populations act as a reservoir of genetic variation within any population. Domestic guinea fowl populations revealed lower genetic diversity. On the other hand, wild populations were genetically more diverse than their domestic counterparts. This is linked to their effective population sizes. ITAD and CHID were moderately diverse among domesticated guinea fowl populations.

The findings from elucidation of ROHs across various populations were consistent with Shen et al. [43], who reported the Italian and Sudanese populations to show the highest mean ROH numbers. The average SNP count per ROH fragment followed a similar pattern, from 1207.79 in SUDW to 5972.34 in SUDD, with Indian guinea fowl (CARI) showing an intermediate estimate of 4110.06. Indian guinea fowl (CARI) showed 62.76% of short ROH (<500 kb), indicating primarily historical inbreeding. These results aligned with findings of Shen et al. [43], who reported wild guinea fowl populations with a lower mean number and total length of ROHs than domestic guinea fowl populations. Among the common associated genes under ROHs across populations, several key candidate genes were identified, including *FOS*, *HMOX*, *MAPK6*, *GPX3*, *IL20RA*, *IFNGR1*, *DNAJB4*, *EPAS1*, *IL20RA*, *IL22RA2*, *IFNGR1*, *CSF1R*, *SOCS5*, *BATF*, *FAS*, and *CD74.* The *FOS* gene in poultry has been reported to play a crucial role in regulating cell growth, immune response, and stress adaptation through control of gene expression as part of the AP-1 transcription factor complex [44]. Similarly, the gene Endothelial *PAS* Domain Protein 1 (*EPAS1*) may contribute to heat stress adaptation by regulating the hypoxia response and angiogenesis, thereby enhancing oxygen delivery and improving cellular resilience [45]. The *CD74* gene is reported to facilitate MHC class II antigen presentation, which is considered essential for mounting effective adaptive immune responses against pathogens in poultry [46,47]. Moreover, *Colony Stimulating Factor 1 Receptor* (*CSF1R*) supports innate immunity by regulating macrophage development and function, thereby playing a crucial role in defending poultry against infections [47]. The variation in the number of ROH among wild and domestic guinea fowl populations was indicative of a signature of domestication and evolutionary dynamics among them. The highest number and length of ROH in IRAD and SUDD, respectively, were indicative of their recent inbreeding. The Indian guinea fowl (CARI) showed intermediate inbreeding as evidenced by a sustained number of ROH and moderate length. Shorter ROH segments dominated the genetic landscape across all populations. Longer segments were indicative of recent inbreeding, as longer segments were not broken by recombination. ROH estimates in wild guinea fowl indicated their distant inbreeding; SUDW was the least inbred population, with only 337 ROH fragments. The findings of the ROH analysis correlated with F_ST_ findings, revealing high genetic differentiation and low diversity, especially for IRAD and ITAD populations.

The largest genomic region covered by a single CNVR was observed in Indian guinea fowl (CARI) (2.16 Mb, representing 0.21 percent of the genome), followed by NIGW (0.16 percent) and NIGD (0.15 percent). Most CNVRs were located on the first five autosomes, while chromosomes 8, 15, 18, 23, and 31 contained the fewest (Table 3). The Indian guinea fowl population (CARI) showed the lowest number of structural variants with the least number of CNVRs. Deletions were primary structure variants in the guinea fowl genome. CNVRs covered a significant portion of the guinea fowl genome. Overall, deletions were a diverse feature of both domestic and wild guinea fowl populations. Several genes harbored under CNVRs were common across populations (Figure 3c), with 1080 genes shared among Indian guinea fowl (CARI), IRAD, and CHID within CNVRs (Figure 3d). Key candidates include *HSPB1*, *HSPA4*, *NRF1*, *CAT*, *BDNF*, *GPX1*, *SLC2A8*, *IL6*, *IFNG*, *IL17RE*, *IL17RC*, *DUSP1*, *CX3CL1*, *CD74*, *TLR4*, *TNFRSF13B/C*, *IRF1*, *IRF8*, *GATA3*, and *NFKB1*, many of which are known to influence heat tolerance and immune defense in poultry. The Heat Shock Protein Beta-1 (*HSPB1*) gene has been reported to help protect cells from heat-induced damage by stabilizing proteins and reducing oxidative stress while also supporting immune function through anti-inflammatory regulation [48]. Similarly, the catalase (*CAT*) gene encodes an enzyme that has been reported to help degrade hydrogen peroxide, protecting poultry cells from oxidative damage caused by heat exposure [49]. Another gene, i.e., Brain-Derived Neurotrophic Factor *(BDNF)*, has been reported to enhance heat resistance by activating the TrkB–MAPK/ERK and PI3K/Akt pathways to protect neurons, while heat-induced promoter demethylation can boost its expression and improve thermotolerance [50]. The Interleukin-6 *(IL6)* gene encodes a pro-inflammatory cytokine that mediates immune responses and helps protect against stress-induced damage; under heat stress, it regulates inflammation, modulates immune cell activity, and supports recovery [51,52]. Another gene, i.e., Dual Specificity Phosphatase 1 *(DUSP1)*, regulates the *MAPK* signaling pathway, affecting immune responses and stress adaptation. Its expression has been reported to be upregulated in heat-stressed chickens, enhancing antigen presentation and immune function [52]. Similarly, the Toll-like Receptor 4 (*TLR4)* gene is a crucial immune receptor in poultry that has been reported to help recognize lipopolysaccharides (LPS) from Gram-negative bacteria, activating innate immune responses and stimulating proinflammatory cytokine production [49].

Guinea fowl have adapted to tropical and semi-arid regions through heat tolerance and disease resilience. They possess strong immune responses to combat pathogens, while thermoregulatory processes and genetic adaptations enable survival in extreme temperatures. Among the genes harbored under intra-population selection sweeps, the B-cell lymphoma/leukemia 11B (*BCL11B*) gene, mapped on NumMel_5, has been reported to promote cellular apoptosis while inhibiting the replication of avian leukosis virus subgroup J (ALV-J) in chickens [53]. Similarly, the Glypican 3 (*GPC3*) gene, mapped on NumMel_8, has been associated with immune traits in white leghorn chickens and has been shown to inhibit hepatocellular carcinoma cells [54,55], indicating its dual role in immunity and tumor suppression. In addition, the immunoglobulin superfamily DCC subclass member 4 gene (*IGDCC4*), mapped on NumMel_9, has been reported to be involved in transmembrane transport and acts as a host factor facilitating the internalization of the H5N1 influenza virus in poultry and humans [56]. Knockout of this gene has been reported to significantly reduce intracellular viral load [56]. Likewise, the Immunoglobulin Superfamily Member 11 (*IGSF11*) gene, on NumMel_1, has been linked to spleen immune responses post-immunization, and its upregulation is associated with enhanced immunity and increased disease resistance in chicken [57]. Additionally, Interleukin-18 (*IL-18*), mapped on NumMel_23, may play a crucial role in the development of T-helper 1 cells [58] and enhances CD4+ and CD8+ T cell responses as well as natural killer (NK) cell activity [59]. On the other hand, the insulin receptor substrate 4 (*IRS4I*) gene, located on NumMel_8, which regulates glycol-metabolism, has been reported to be significantly downregulated in chickens infected with reticulo-endotheliosis virus [60]. Moreover, the Transcription Factor 12 (*TCF12*) gene, mapped on NumMel_9, has been reported to play a vital role in B- and T-cell development [61] and shows suppressed expression in Marek’s disease-susceptible chicken lines [62]. In addition, the Ten-Eleven Translocation Methylcytosine Dioxygenase 3 (*TET3*) gene, mapped on NumMel_21, may be important for immunoglobulin diversification via modulation of non-CpG methylation in immunoglobulin pseudogenes [63]. On the other hand, Cadherin-13 (*CDH13*), located on NumMel_10, has been implicated in resistance to *Campylobacter jejuni* colonization in poultry [64]. Collectively, these genes highlight multiple immune-related mechanisms potentially under selection in Indian guinea fowl, reflecting their adaptation to pathogen-rich environments. In addition to immune resilience, genes like *BAG3* and *TRPV4* contribute to heat stress adaptation in poultry birds. The BAG cochaperone 3 (*BAG3*) gene, located on NumMel_4, may support thermoregulation besides improving heat tolerance and performance [65]. Another gene, i.e., *TRPV4* (transient receptor potential cation channel subfamily V member 4), a heat-sensitive ion channel on NumMel_14, has been reported to help regulate key responses to thermal stress. It has been reported to be involved in ameliorating intestinal inflammation via embryonic thermal manipulation [66]. Together, these genes highlight key molecular pathways involved in enhancing heat resilience in poultry.

Several other genes were identified that were associated with fat deposition and skin color in poultry. The Acetoacetyl-CoA synthetase (*AACS*) gene, mapped on NumMel_14, has been reported to be involved in converting acetoacetate to acetoacetyl-CoA, enabling the incorporation of ketone bodies into cholesterol and fatty acids, and is highly expressed in lipogenic tissues such as the liver, brain, and adipose [67,68]. The Actin Gamma 2 (*ACTG2*) gene, located on NumMel_21, has been reported to inhibit pre-adipocyte differentiation, thereby regulating fat cell development and energy balance [69]. Additionally, the *ELOVL4* (elongation of very long chain fatty acids-4) gene, mapped on NumMel_3, contributes to the synthesis of very long-chain fatty acids (VLCFAs) and plays a key role in intramuscular fat content and overall lipid distribution [70]. The *BCO2* (β-carotene oxygenase 2) gene, mapped on NumMel_23, may help regulate skin color in poultry by breaking down yellow carotenoids into colorless compounds. Its downregulation or mutation has been reported to cause carotenoid buildup, resulting in yellow skin, while active expression leads to white or pale skin [71]. The present study identified candidate genes in guinea fowl and related species (wild counterparts) that play crucial roles in immune defense, thermal adaptation, fat metabolism, and pigmentation. These genetic variants have likely undergone selective sweeps, enhancing the environmental resilience and local adaptation of the Indian guinea fowl populations.

The genes that were found to overlap with selection sweep under the comparison of Indian guinea fowl with other domesticated counterparts within the analyzed genomic windows included *STAU2*, *TCF7*, *CPQ*, *CTNNB1*, *ECI2*, *GRID2*, and *ACSS2*. The *CHMP4B* (charged multivesicular body protein 4B) gene, located on NumMel_19, has been reported to facilitate Newcastle Disease Virus (NDV) replication by promoting viral budding [72], while the *CHCHD10* (coiled-coil-helix-coiled-coil-helix domain-containing protein 10) gene on NumMel_14 has been associated with host defense during NDV infection by regulating mitochondrial activity and interferon signaling [73]. The *TNFRSF21* (Tumor Necrosis Factor Receptor Superfamily Member 21) gene, mapped on NumMel_3, may contribute to immune defense and apoptosis in poultry and shows increased expression during viral infections, including fowl adenovirus and potentially NDV [74]. Additionally, the *ROBO2* (roundabout axon guidance receptor homolog 2) gene on NumMel_1 has been associated with the antibody response to NDV, besides influencing ovarian follicle development through the SLIT-ROBO signaling pathway [75]. These genes collectively highlight essential mechanisms of host–virus interaction, immune activation, and reproductive resilience against NDV in poultry.

The *STAU2* (Staufen double-stranded RNA binding protein 2) gene, mapped on NumMel_2, has been reported to play a critical role in avian influenza virus (AIV) infection in poultry by interacting with the viral NS1 protein, thereby promoting viral replication and modulating host–virus interactions [76]. Further emphasizing immune response, the *TNFRSF21* (Tumor Necrosis Factor Receptor Superfamily Member 21) gene on NumMel_3 has been reported to enhances immune defense and apoptosis in poultry, particularly under viral challenges such as fowl adenovirus (FAdV-4) infection [77], while *TCF7* (Transcription Factor 7), located on NumMel_12, may promote T-cell activation and gut immunity through the Wnt signaling pathway, contributing to resistance against Marek’s disease [78]. Addressing cardiovascular health, the *CPQ* (Carboxypeptidase Q) gene, also mapped on NumMel_2, has been reported to play a vital role in broiler health by influencing vascular function and blood pressure regulation, and has been associated with ascites syndrome, a condition related to pulmonary hypertension [79]. The *C2CD5* (C2 calcium-dependent domain-containing 5) gene on NumMel_1 encodes a protein involved in membrane trafficking and signal transduction and is considered essential in mounting an immune response against Salmonella infections, with additional roles in viral defense, alternative splicing, and traits linked to domestication [80]. Together, these genes form a crucial network governing immune competence, viral and bacterial resistance in poultry.

Consistent with the results obtained through the iHS method, several genes identified within the selection signals in this comparison were associated with heat tolerance traits in guinea fowl. The *ECI2* (enoyl-CoA delta isomerase 2) gene on NumMel_2 has been reported to play a vital role in mitochondrial β-oxidation of unsaturated fatty acids and is consistently upregulated under heat stress conditions, with its expression in liver and muscle supporting energy metabolism and contributing to heat tolerance and lipid regulation in poultry [81]. Similarly, the *GRID2* (Glutamate Ionotropic Receptor Delta Type Subunit 2) gene, also located on NumMel_2, has been identified as a candidate gene involved in poultry adaptation to extreme temperature conditions [82]. In addition, the *ACSS2* (acyl-CoA synthetase short-chain family member 2) gene, mapped on NumMel_19, is considered critical for converting acetate into acetyl-CoA, thereby influencing lipogenesis, energy metabolism, and fat deposition, particularly under dietary or thermal stress. It has also been reported to play an important role in gut-liver axis signaling and has been associated with meat quality traits in both broilers and layers [67]. Supporting these functions, the *LGR4* (leucine-rich repeat containing G protein-coupled receptor 4) gene on NumMel_5 may contribute to thermal adaptation by regulating cellular signaling and stress response mechanisms, with varying expression levels observed between heat- and cold-tolerant poultry breeds, further supporting its involvement in temperature adaptation [83]. These genes collectively support enhanced heat tolerance and metabolic adaptation in poultry.

The *ACAD9* (acyl-CoA dehydrogenase family member 9) gene on NumMel_11 is considered crucial for mitochondrial function and fatty acid β-oxidation in chickens, serving as a key biomarker for intramuscular fat deposition [84]. Similarly, the *CTNNB1* gene, which encodes β-catenin and is mapped on NumMel_2, has been reported to play a central role in the Wnt/β-catenin signaling pathway, contributing to reproductive efficiency [85], feather development [86], and adipogenesis [87] across various poultry species. Supporting lipid metabolism, the *ME3* (malic enzyme 3, NADP-dependent, mitochondrial) gene on NumMel_11 has been reported to facilitate energy regulation by producing NADPH, with its expression notably increased under cold stress and threonine deficiency, thereby linking it to both climate adaptation and nutrient response [88]. Complementing these functions, the *ATRNL1* (Attractin-like 1) gene, located on NumMel_4, has emerged as a candidate gene for feed efficiency, as genome-wide studies have suggested its involvement in regulating energy metabolism and neuronal pathways that influence feed intake and conversion efficiency [89]. In line with feed regulation, the *SPX* gene, encoding Spexin and mapped on NumMel_1, has been reported to act as a neuropeptide that suppresses appetite by targeting the hypothalamus, thus regulating the feeding behavior and serving as a potential target for improving growth efficiency in poultry [90].

The manuscript reports the first attempt at delineating various aspects of adaptation of global domesticated and wild cohorts of guinea fowl populations using WGS data. The limited sample size of Indian domestic guinea fowl (CARI) may have limited the statistical power of the analysis, especially the iHS component, while the rare variants may have been under-represented. However, higher coverage of the genome was available for the analysis. The CNV calling procedures were undertaken on a per-sample basis along with their bin-size standardization and filtering. The functionality of various genes was mostly inferred from chicken-based studies, as the same are not extensively available for guinea fowl populations. Based on these points, the results may be considered putative in nature, pending further experimental validation and integration with phenotype or functional data.

## 4. Materials and Methods

### 4.1. Sampling

Six Pearl variety guinea fowl (3 adult male and 3 female birds) were randomly chosen from the experimental farm of ICAR-Central Avian Research Institute (CARI), Bareilly, India. Blood samples were collected from the jugular vein following the guidelines of the Institutional Animal Ethics Committee (IAEC) of CARI. Genomic DNA was isolated using the Qiagen DNeasy^®^ Blood (Thermo Fisher Scientific, Waltham, MA, USA) and Tissue Kit. The isolated DNA samples were evaluated (with blinding) for quality as well as quantity using a standard spectrophotometer and agarose gel electrophoresis documentation procedures. Next-generation sequencing (NGS) libraries were constructed using the NEBNext^®^ Ultra^TM^ DNA Library Prep Kit (Illumina^®^, San Diego, CA, USA). Sequencing was undertaken on the Illumina^®^ NovaSeq 6000 platform at 20× coverage with 150 bp paired-end reads at AgriGenome Labs Pvt. Ltd., Kerala, India, with blinding.

**Figure 7:**
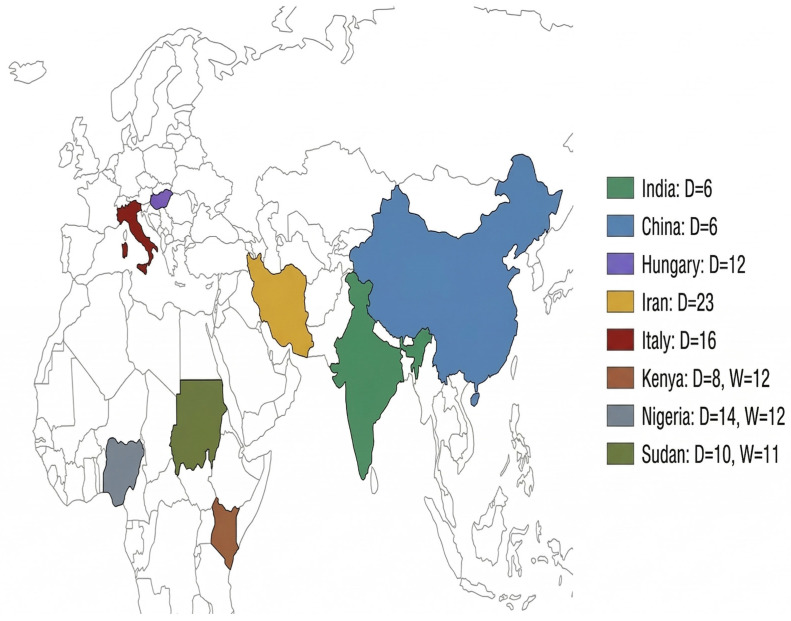
Map showing sampling locations for domestic and wild guinea fowl (D implies domestic, W refers to wild populations).

Employing the concept of data reuse in agriculture and allied sciences, the WGS data on 124 other guinea fowl genomes representing domestic and wild lineage across 10 populations were also retrieved and used (PRJNA639777) [41]. This included 89 domestic guinea fowl samples, sourced from Iran (IRAD, *n* = 23), China (CHID, *n* = 6), Italy (ITAD, *n* = 16), Hungary (HUGD, *n* = 12), Sudan (SUDD, *n* = 10), Kenya (KEND, *n* = 8), and Nigeria (NIGD, *n* = 14). Additionally, to enable a comprehensive assessment of genetic diversity and evolutionary dynamics, WGS data from an additional 35 wild guinea fowl were incorporated in the study. These wild samples provided additional dimensionality to the study with representation from Nigeria (NIGW, *n* = 12), Kenya (KENW, *n* = 12), and Sudan (SUDW, *n* = 11). The geographical and ecological distribution of various populations analyzed in the present study is depicted in Figure 7.

### 4.2. Read Alignment, Post-Alignment Processing, and Variant Calling

Quality assessment of the raw data was conducted using FastQC v0.12.0 (https://www.bioinformatics.babraham.ac.uk/projects/fastqc/; accessed: 15 April 2025). Adapter sequences and low-quality reads were removed using TrimGalore [91,92], followed by re-evaluation with FastQC. After filtering and genome indexing, reads from domestic and wild helmeted guinea fowl were aligned to the latest guinea fowl reference genome (NumMel1.0: GCA_002078875.2), using Burrows-Wheeler Aligner (BWA, v0.7.17) with default settings [93]. The genome assembly (GCA-002078875.2; release: 12 January 2017) for helmeted guinea fowl is 1.0433 Gb (1,043,264,150 b) across 32 assembled chromosomes, sex chromosome (Z), mitochondria, and 2428 scaffolds. Chromosomes 28, 29, and 30 are not assembled or unambiguously placed as scaffolds. The number of annotated genes in this genome is 21,846, with 16,083 protein-coding genes.

Sequence alignment map (SAM) files were converted to binary format binary alignment map (BAM) using SAMtools v1.20 [94]. Subsequently, the PCR duplicates in BAM files were identified using the MarkDuplicates function of Picard tools v2.25.1 (https://broadinstitute.github.io/picard/, accessed: 18 April 2025). Variant calling was performed using Genome Analysis ToolKit (GATK v4.4.0.0; https://software.broadinstitute.org/gatk, accessed: 18 April 2025) with the HaplotypeCaller to reconstruct haplotypes and identify SNPs and InDels. The CombineVCFs tool was used to merge multiple GVCF files. Subsequently, the GenotypeGVCFs function of GATK toolkit was used to convert GVCFs into standard VCFs containing information on genome-wide markers. Finally, SelectVariants was used to extract SNP subsets from the VCF file.

### 4.3. Quality Control (QC) of Genome-Wide SNP Data

The genetic variants were initially filtered using the VariantFiltration tool within GATK to ensure high-confidence markers and reliable predictions. The filtration thresholds included a quality depth (QD) score below 2, a Phred-scaled genotype quality (QUAL) score below 30, a mapping quality (MQ) score under 40, a Fisher strand bias score above 60, a SOR greater than 3, a mapping quality rank sum test (MQRankSum) score below −12.5, and a Read Position Rank Sum Test (ReadPosRankSum) score below −8. The dataset was further processed for additional QC thresholds in the PLINK program v1.9 [95], excluding SNP markers lacking coordinates on the reference genome, sex chromosomes, or mitochondrial DNA. Further, the standard thresholds for genotyping rate, call rate, minor allele frequency, Hardy–Weinberg equilibrium, and Mendelian errors were employed in the PLINK program.

### 4.4. Population Structure Analysis

Breed clustering and population structure among the 11 guinea fowl cohorts were assessed using ADMIXTURE v1.3 [96]. On the other hand, principal component analysis (PCA) was undertaken with genome-wide complex trait analysis (GCTA) v1.93 [97], generating a genomic relationship matrix (GRM) from binary PLINK files, with the top three principal components visualized using the ggplot2 package [98] in the R-programming environment. Similarly, the phylogenetic relationships, based on a distance matrix from PLINK, among individuals across 11 populations were constructed using the neighbor-joining (NJ) method in MEGA11 [99]. The resulting NJ tree was visualized using an online tool, i.e., iTOL [100].

The degree of genetic differentiation was quantified using F_ST_ for 55 pairwise population comparisons, with values ranging from 0 (no differentiation) to 1 (complete differentiation). VCFtools [94] was used to calculate Wright’s fixation index (F_ST_) and nucleotide diversity (π) with a 100 kb sliding window and 10 kb step. The genetic diversity of the guinea fowl populations was further assessed using minor allele frequency (MAF) empirical distribution across five bins: I (<0.10), II (0.10–0.20), III (0.20–0.30), IV (0.30–0.40), and V (>0.40). MAF was calculated in PLINK, and customized scripts determined marker proportions across bins. Linkage disequilibrium (r^2^) was also assessed in PLINK across 11 populations.

### 4.5. Runs of Homozygosity (ROH)

ROH fragments were detected in each population separately using PLINK program with the following settings and flags: *--homozyg-gap 1000* (maximum physical gap in kb allowed between two consecutive homozygous SNPs; *--homozyg-kb 250* (minimum length of an ROH in kilobases); *--homozyg-snp 50* (minimum number of SNPs required for an ROH); *--homozyg-window-het 3* (number of heterozygous calls within an ROH); *--homozyg-window-missing* 5 (scanning window can contain up to 5 missing calls); *--homozyg-window-threshold 0.05* (minimum proportion of homozygous SNPs a sliding window must contain to be considered a ‘hit’); and *--homozyg-window-snp 50* (size of this window in terms of the number of SNP). The parameters were standardized as per Meyermans et al. [101]. ROHs were grouped into four length categories (<0.5 Mb, 0.5–1 Mb, 1–2 Mb, >2 Mb).

### 4.6. CNV Detection, Filtering, and Concatenation

The CNVs along the genome of guinea fowl populations were elucidated using CNVnator v0.4.1 [102]. It uses a read-depth-based tool that divides the genome into user-defined non-overlapping bins and calculates read depth (RD) to identify CNVs. Optimal bin size for each sample, selected as a multiple of 100, was determined based on read depth, length, and variance, maintaining a recommended RD:variance ratio of 4–5 [102]. GC bias was corrected within CNVnator using aligned bam files, and the information from the reference genome, and CNVs were called using the ‘call’ function. Post CNV detection, quality control, and filtering were performed based on *p*-value (<0.01), mapping quality (<0.5), CNV size (>1 kb), and q0 (zero mapping quality fraction < 0.5). Overlapping CNVs (≥1 bp) were merged into CNV regions (CNVRs) using a custom Python v3.13 script and categorized as deletions, duplications, or mixed type. The RIdeogram R-package was used to visualize CNVR distribution along different chromosomes with relation to gene density, with gene annotations extracted from the GFF file, with visualization under a 1 Mb window.

### 4.7. Selection Signature Analysis

Selection signatures in Indian guinea fowl were investigated using two complementary approaches, i.e., intra-population analysis using the haplotype-based iHS statistic [103] and inter-population analysis with cross-population extended haplotype homozygosity (XP-EHH), to ensure a comprehensive assessment of both within- and across-population selection patterns. Under the iHS analysis, VCF files were phased using the BEAGLE v5.5 program [104] and processed in the Selscan v3.0 [105], where SNPs with MAF ≤ 0.01 were excluded, scores were standardized within allele frequency bins, and averaged over 50 kb non-overlapping windows. The XP-EHH analysis used the same phased VCF dataset in Selscan v3.0, with Indian guinea fowl set as the observed population and domestic guinea fowl as the reference, calculating XP-EHH scores as log ratios of integrated EHH, standardizing them, and identifying significant signals through Manhattan plot visualization. The top 1% of the signals were considered as putative sweeps for the downstream analysis.

### 4.8. Gene Annotation of Regions Under Selection

Overlaps between CNVRs and genes identified from ROH, CNVR analyses, and across different populations were analyzed. Information on ROH, CNV, and selection signatures was obtained from relevant databases. Candidate regions under selection were analyzed for gene functions using database mining. Genes within these regions were annotated with the GenomicRanges R package [106] and a customized package to identify overlaps with known QTLs and traits. Functional enrichment was performed using DAVID [107], focusing on Gene Ontology (GO) terms and Kyoto Encyclopedia of Genes and Genomes (KEGG) pathways [108] to identify key biological processes and pathways associated with the candidate genes.

## 5. Conclusions

This study presents the first global CNV atlas for guinea fowl and provides the first comprehensive genomic characterization of the Indian domestic population, integrating ROH, CNV, and selection signature analyses. Our findings revealed that Indian guinea fowl share close genetic affinities with Iranian and Chinese populations yet retain distinct signatures shaped by domestication and selection. Importantly, the overlap of ROH, CNVR, and selective sweeps with functionally relevant genes underscores the genomic mechanisms that enable guinea fowl to thrive under heat stress and pathogen-rich environments. These insights not only advance our understanding of guinea fowl domestication and adaptation but also provide a valuable genomic resource for conservation and genetic improvement strategies aimed at enhancing climate resilience in poultry production. Collectively, this work positions guinea fowl as a model species for studying genetic resilience to environmental stressors, with direct implications for sustainable poultry farming in the face of global climate change.

## Figures and Tables

**Table 1:** The minor allele frequency (MAF) empirical distribution patterns across five different bins and LD estimates in 11 guinea fowl populations.

MAF Bin	x < 0.1	0.1 ≤ x < 0.2	0.2 ≤ x < 0.3	0.3 ≤ x < 0.4	x > 0.4	LD (r^2^)
CARI	5,729,113 (66.44%)	652,140 (7.56%)	653,248 (7.58%)	630,086 (7.31%)	957,876 (11.11%)	0.14
IRAD	4,450,482 (51.61%)	1,189,340 (13.79%)	1,037,360 (12.03%)	934,833 (10.84%)	1,010,448 (11.72%)	0.18
CHID	4,943,260 (57.33%)	901,921 (10.46%)	795,783 (9.23%)	791,128 (9.18%)	1,190,371 (13.81%)	0.19
ITAD	4,892,300 (56.74%)	910,816 (10.56%)	886,717 (10.28%)	895,913 (10.39%)	1,036,717 (12.02%)	0.17
HUGD	3,996,721 (46.35%)	1,282,100 (14.87%)	1,106,564 (12.83%)	1,010,010 (11.71%)	1,227,068 (14.23%)	0.20
SUDD	4,599,974 (53.35%)	1,209,467 (14.03%)	578,228 (6.71%)	1,202,252 (13.94%)	1,032,542 (11.98%)	0.20
KEND	4,226,125 (49.01%)	1,520,059 (17.63%)	712,953 (8.27%)	1,208,480 (14.02%)	954,846 (11.07%)	0.20
NIGD	3,937,957 (45.67%)	1,436,769 (16.66%)	1,203,375 (13.96%)	1,112,605 (12.90%)	931,757 (10.81%)	0.21
NIGW	3,146,509 (36.49%)	1,590,794 (18.45%)	1,766,416 (20.49%)	983,615 (11.41%)	1,135,129 (13.16%)	0.21
SUDW	3,856,388 (44.72%)	1,177,750 (13.66%)	1,120,137 (12.99%)	1,095,315 (12.70%)	1,372,873 (15.92%)	0.25
KENW	3,926,939 (45.54%)	878,140 (10.18%)	1,132,591 (13.14%)	1,316,132 (15.26%)	1,368,661 (15.87%)	0.22

**Table 2:** ROH profile: number, size, SNPs, and density along with genomic coverage across 11 guinea fowl populations.

	CARI	IRAD	CHID	ITAD	HUGD	SUDD	KEND	NIGD	NIGW	SUDW	KENW
TOTAL											
No.	2983	10,552	2746	9678	4689	4787	3446	1384	5218	337	3268
Size (kb)	547.62	585.96	585.46	582.67	622.33	719.08	650.11	563.52	515.9	363.69	405.49
Average SNP	4110.06	4391.32	4391.23	4258.17	3050.56	5972.34	2640.05	3454.09	2960.98	1207.79	2640.56
Average density	0.18	0.18	0.19	0.19	0.18	0.18	0.19	0.2	0.23	0.34	0.21
<500 kb											
No.	1872	5931	1472	5561	2573	2262	1840	928	4760	301	2642
%	62.76	56.21	53.61	57.46	54.87	47.25	53.40	67.05	91.22	89.32	80.84
Size (kb)	348.2	352.24	352.2	381.89	350.9	363.71	352.62	342.87	320.45	320.13	334.6
Average SNP	3008.9	3032.92	2710.04	2909.09	2829.76	3108.92	2875.62	2444.66	1065.35	1041.45	2300.12
Average density	0.17	0.18	0.19	0.18	0.18	0.19	0.18	0.2	0.24	0.34	0.21
500–1000 kb											
No.	816	3418	877	3029	1471	1633	1073	312	347	31	545
%	27.36	32.39	31.94	31.30	31.37	34.11	31.14	22.54	6.65	9.20	16.68
Size (kb)	675.79	685.26	690.83	689.18	684.34	696.03	691	679.42	682.41	642.08	630.38
Average SNP	4899.06	5073.05	4509.01	4824.31	4930.08	5877.27	4734.61	4011.83	3872.73	2145.32	3700.9
Average density	0.19	0.18	0.2	0.2	0.19	0.18	0.2	0.21	0.22	0.32	0.22
1001–2000 kb											
No.	259	1091	348	953	697	493	441	40	96	5	68
%	8.68	10.34	12.67	9.85	14.86	10.30	12.80	2.89	1.84	1.48	2.08
Size (kb)	1257.55	1305.98	1324.44	1310.98	1344.11	1346.78	1310.98	1323.44	1285.65	1240.18	1236.56
Average SNP	8089.66	8119.34	7886.45	8581.79	9051.23	10,507.76	8774.9	6929.56	7109.87	5368.4	6910.32
Average density	0.2	0.2	0.21	0.2	0.2	0.17	0.19	0.22	0.22	0.27	0.23
>2000 kb											
No.	38	129	51	126	99	197	94	14	13	0	4
%	1.27	1.22	1.86	1.30	2.11	4.12	2.73	1.01	0.25	0.00	0.12
Size (kb)	2514.06	2639.13	2661.54	2709.04	2763.34	2900.86	2909.87	2543.09	2767.45	0	2611.13
Average SNP	14,297.89	17,444.31	16,768.88	17,953.09	21,392.65	23,576.67	19,877.76	13,343	14,163.65	0	11,048.75
Average density	0.2	0.19	0.19	0.19	0.17	0.16	0.18	0.22	0.21	0	0.24

**Table 3:** Summary statistics of CNVR counts, types, and genomic coverage across 11 guinea fowl populations.

Breed	CNVR	Total Length	Deletion	Duplication	Mixed	Largest CNVR	Genomic Coverage	Z	MAX		MIN	
									Chr	No.	Chr	No.
CARI	1508	193,362,184	1266	208	34	2,261,999	18.53	138	1	270	31	3
IRAD	4024	76,373,876	3783	221	20	1,018,099	7.32	551	1	632	31	3
CHID	3081	74,601,419	2997	78	6	1,333,599	7.15	427	1	472	31	5
ITAD	4063	122,302,937	3634	336	93	1,141,399	11.72	403	1	677	31	4
HUGD	6633	77,826,367	6496	123	14	977,899	7.46	778	1	1412	31	3
SUDD	3703	72,510,497	3568	126	9	1,017,599	6.95	570	1	591	31	2
KEND	2654	75,811,746	2564	82	8	1,309,999	7.27	385	1	417	31	3
NIGD	2559	72,485,141	2474	75	10	1,674,999	6.95	373	1	427	31	2
NIGW	2595	64,730,405	2465	120	10	1,699,499	6.20	366	1	435	31	2
SUDW	4220	65,585,380	4036	172	12	1,017,899	6.29	584	1	715	31	4
KENW	5027	64,220,573	4789	226	12	940,599	6.16	677	1	956	31	3

## Data Availability

The raw data on guinea fowl populations are available in NCBI with accession No. PRJNA639777.

## Supplementary Materials

The following supporting information can be downloaded at: https://www.mdpi.com/article/10.3390/ijms27072994/s1.
