# Supplementary material for: Comprehensive Analysis of 122 Guinea Fowl Genomes Across Three Continents Delineates Their Domestication and Evolutionary Patterns with Special Reference to India
Source: Int J Mol Sci. 2026 Mar 25;27(7):2994. doi: 10.3390/ijms27072994 (PMC13073949; doi:10.3390/ijms27072994)
Supplement: Supplementary file 1 [file ijms-27-02994-s001.zip › S1_MAF.pdf]

Summary of the SNP markers at whole-genome scale along with average minor allele frequency (MAF) estimates for each autosomal chromosome (NumMel) in Indian Guinea fowl

Chromosome	Chromosome size (length in bp)	No. of SNP Markers	Average SNP interval (length in bp)	Average MAF
NumMel_1	19,44,37,802	14,36,739	135.33	0.205963
NumMel_2	14,71,28,157	9,44,042	155.85	0.203048
NumMel_3	11,20,45,409	7,51,330	149.13	0.208466
NumMel_4	9,74,84,820	7,05,255	138.23	0.206585
NumMel_5	7,15,86,700	5,56,554	128.62	0.212559
NumMel_6	6,11,78,980	5,38,854	113.54	0.208619
NumMel_7	3,01,84,877	3,16,296	95.43	0.21307
NumMel_8	1,95,49,463	2,54,734	76.74	0.215313
NumMel_9	2,01,92,527	2,54,647	79.30	0.211048
NumMel_10	1,97,63,385	2,26,423	87.29	0.214946
NumMel_11	2,00,42,104	2,68,160	74.74	0.215054
NumMel_12	1,78,92,257	2,69,167	66.47	0.214679
NumMel_13	1,55,49,469	2,58,760	60.09	0.218743
NumMel_14	1,31,98,683	2,12,595	62.08	0.219267
NumMel_15	2,52,168	8,023	31.43	0.220572
NumMel_16	1,03,68,520	1,84,428	56.22	0.216412
NumMel_17	1,06,43,954	1,88,949	56.33	0.218678
NumMel_18	1,02,19,231	1,81,828	56.20	0.219376
NumMel_19	1,40,19,296	2,14,548	65.34	0.218986
NumMel_20	70,00,625	1,34,199	52.17	0.219039
NumMel_21	46,85,633	88,313	53.06	0.222229
NumMel_22	59,86,824	1,18,886	50.36	0.219784
NumMel_23	65,29,417	1,24,077	52.62	0.219212
NumMel_24	21,43,333	41,301	51.90	0.217383
NumMel_25	54,95,279	1,17,551	46.75	0.218061
NumMel_26	47,68,521	98,793	48.27	0.21881
NumMel_27	50,69,901	99,692	50.86	0.216758
NumMel_31	1,10,790	1,606	68.99	0.216942
NumMel_32	14,43,594	26,713	54.04	0.216882
